# RECOT: a tool for the coordinate transformation of next-generation sequencing reads for comparative genomics and transcriptomics

**DOI:** 10.1186/1751-0473-8-6

**Published:** 2013-02-26

**Authors:** Akiko Izawa, Jun Sese

**Affiliations:** 1 Department of Computer Science, Ochanomizu University, Tokyo, Japan; 2 Department of Computer Science, Tokyo Institute of Technology, Tokyo, Japan

**Keywords:** Next-generation sequencers, RNA-seq, ChIP-seq, Comparative genomics, Comparative transcriptomics

## Abstract

**Background:**

The whole-genome sequences of many non-model organisms have recently been determined. Using these genome sequences, next-generation sequencing based experiments such as RNA-seq and ChIP-seq have been performed and comparisons of the experiments between related species have provided new knowledge about evolution and biological processes. Although these comparisons require transformation of the genome coordinates of the reads between the species, current software tools are not suitable to convert the massive numbers of reads to the corresponding coordinates of other species’ genomes.

**Results:**

Here, we introduce a set of programs, called REad COordinate Transformer (RECOT), created to transform the coordinates of short reads obtained from the genome of a query species being studied to that of a comparison target species after aligning the query and target gene/genome sequences. RECOT generates output in SAM format that can be viewed using recent genome browsers capable of displaying next-generation sequencing data.

**Conclusions:**

We demonstrate the usefulness of RECOT in comparing ChIP-seq results between two closely-related fruit flies. The results indicate position changes of a transcription factor binding site caused sequence polymorphisms at the binding site.

## Background

Recent and ongoing advances in DNA sequencing technologies have permitted the genome sequences of non-model animals and plants to be determined. Now that the genome sequences are available, various experiments using these sequences, such as RNA-seq [1] and ChIP-seq [2], have been performed. Comparison of the results obtained from non-model organism with data from closely related model organism is an important analysis which enhances our understanding of how genetic polymorphisms contribute to differences in phenotypes such as gene expression and regulation.

There are two different approaches for these comparisons. One approach is to directly align the short reads determined from the query species to the genome of the target species for comparison. The other approach is to match the nucleotides in the genome of the observed species to those of the target species using an alignment program, and then transform the coordinates of the short reads according to the correspondence of the nucleotides between the genomes. The former approach may cause problems when corresponding query and target sequences are dissimilar, as the genetic polymorphism may cause inaccurate or prevent the alignment of the reads to the target genome. To perform the latter approach, a genome conversion tool called liftOver [3] might be useful. However, the program converts an annotation format called BED and cannot handle the standard next-generation sequence formats, SAM and BAM. Therefore, liftOver is not suitable for studying comparative genomics and transcriptomics using next-generation sequencers.

In this paper, we introduce a set of programs, called REad COordinate Transformer (RECOT) created to convert the alignment or mapping coordinates of short reads obtained from the query species (for example a non-model organism) to a comparison target species (for example a model species). After the user inputs the short reads, the query species genome, its gene regions, and the target species genome, our scripts automatically calculate the corresponding regions between the genome sequences and transform the coordinates of the short reads from the query species to the target species. Because RECOT generates an output file in the SAM format, the results can be visualized in most genome browsers that can handle next-generation sequences.

## Implementations

In this section, we describe the features and usage of RECOT. Python is required to run RECOT. We call the species from which the short reads were obtained the *query* species and the species to which the coordinates of the short reads will be transformed for comparison the *target* species.

**Figure 1:**
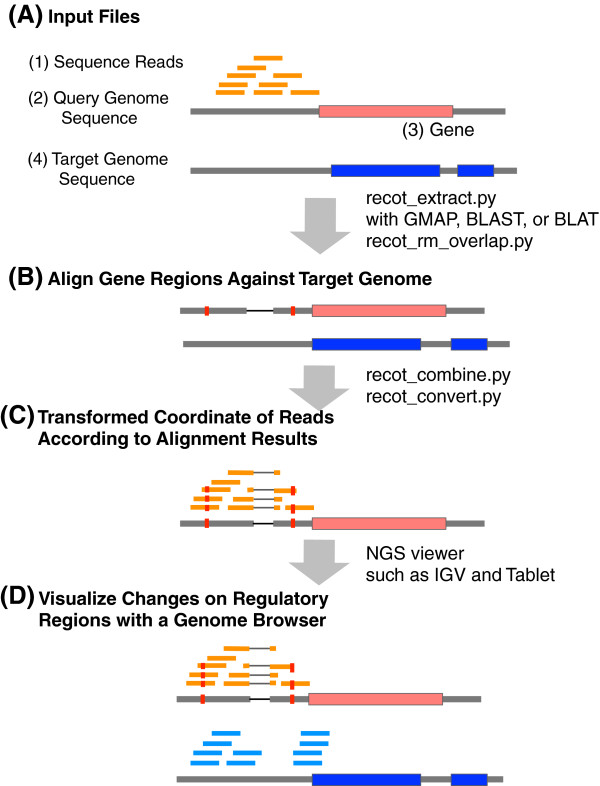
**A schematic overview of read coordinate transformer.** This figure illustrates a comparison of ChIP-seq data between two different species.

RECOT requires four files (Figure 1A): (1) the short reads whose mapping coordinates you want to transform, (2) the genome sequence of the query species, (3) the gene regions of the query genome in the GFF3 format, and (4) the genome sequence of the target species. The next-generation sequencing short reads should be mapped onto the genome sequence of the query species and the results converted into the SAM format. All of the settings used in RECOT, including input and output filenames and parameters, are described in the file “settings.ini”.

The script “recot_extract.py” extract the gene region sequences with their 500-bp upstream sequences from the transcription start sites and downstream sequences from the 3^′^-end. The upstream and downstream lengths are defined in the configuration file. The query sequences need to be aligned to the target species genome to identify their corresponding regions (Figure 1B). Because the output sequences of recot_extract.py are given in FASTA format, you can select a long sequence alignment tool, such as GMAP [4], BLAST [5], or BLAT [6]. If you use BLAST or BLAT for this step, you will need to convert the output to SAM format.

In the alignment results, a target genome region may associate with multiple query gene sequences due to the existence of paralogous genes. To address the problem of such one-to-multi or multi-to-multi results in the query-to-target sequence alignments, we have prepared an optional script that can select sequence relations (1) that are prioritized by user-specified relationships between query and target genes by describing the relations in a text file and (2) highest alignment score, when query and target alignments are not reciprocally unique.

Next, the SAM file of the short reads mapped to the query species genome, using a short read alignment tool such as BWA [7] or bowtie [8], are transformed according to the alignment relationships between the query and target genomes (Figure 1C). For the transformation, we map the differences in the nucleotides between the two species, as calculated in the previous step, onto each read. To accomplish this task, we have prepared two scripts. The first script, “recot_combine.py”, generates temporary files that contain associations between the short read query species mapping positions and the corresponding positions of the target species. The second script, “recot_convert.py”, converts the mapping coordinates of the short reads to the query genome to the corresponding positions of the target genome and generates the transformed SAM file. Reads on the non-corresponding positions are regarded unmapped in the SAM file. The transformation of billions of reads by “recot_convert.py” may take several hours. To accelerate the process, recot_convert.py can run the transformation in parallel, using the job queueing system of Oracle (SUN) Grid Engine or Torque.

These results can be visualized using genome browsers that can handle SAM format, such as IGV [9]. Supplementary Figure 1 is a screen shot of IGV in which ChIP-seq experiments in *D. melanogaster* and *D. simulans* are compared. The result shows the conservation of binding sites, even though the region contains short deletions and several single nucleotide polymorphisms.

RECOT can be used for species in which the genome sequence is unavailable. For these species, we first generate gene sequences using a transcriptome assembly tool such as Trinity [10], and we regard these sequences as genome sequences (we regard each gene/contig as a chromosome). The subsequent procedures are the same as the steps in Figure 1.

## Results

**Figure 2:**
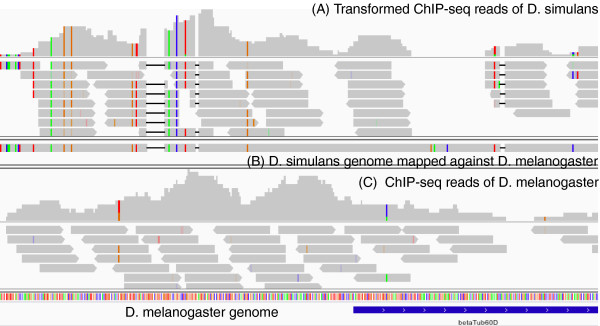
**An example of the coordinate transformation results.** (**A**) The ChIP-seq results from *D. simulans*. The coordinates have been converted into *D. melanogaster* using RECOT. The upper histogram indicates the number of reads associated with the position. The gray horizontal lines are short reads. The colored positions indicate differences from *D. melanogaster* genome. The black narrow bars indicate deletions. (**B**) The *D. simulans* gene regions with 500-bp upstream and downstream sequences. The sequence was mapped onto the *D. melanogaster* genome using GMAP. The result shows us that the non-coding region contains mutations and short deletions. (**C**) The ChIP-seq results from *D. melanogaster* (ERR020066).

We applied RECOT to the comparison of regulatory regions between *D. melanogaster* and *D. simulans* (Figure 2). In Figure 2, the coordinates of ChIP-seq results on *D. simulans* (ERR020078 in DDBJ Sequence Read Archive) [11] are transformed onto the *D. melanogaster* genome [12]. These species diverged approximately 5 million years ago [13]. We can determine from this figure that the transcription factor binding sites identified in the ChIP-seq analysis are similar to each other even though there are deletions and other polymorphisms.

## Conclusions

We developed a set of programs to transform the coordinates of short reads between two species. Our tools can visualize the differences in the genome regions onto which the short reads are mapped between two genomes. Our tools are useful for comparative genomics and comparative transcriptomics between model and non-model organisms.

### Availability and requirements

**Project name**: RECOT project

**Project home page**: http://sesejun.github.com/recot/

**Operating systems**: UNIX, Mac OS X and Windows

**Programming language**: Python

**Other requirements**: Python 2.5 or higher

**Licence**: BSD

**Any restrictions to use by non-academics**: no licenses are required

## Competing interests

The authors declare that they have no competing interest.

## Authors’ contributions

AI and JS designed this research and implemented all the scripts. JS drafted the manuscript. Both authors read and approved the final manuscript.

## Authors’ information

The source codes along with user documentation are available at http://sesejun.github.com/recot/

Contact: sesejun@cs.titech.ac.jp
